# Possible prediction of the response of esophageal squamous cell carcinoma to neoadjuvant chemotherapy based on gene expression profiling

**DOI:** 10.18632/oncotarget.6554

**Published:** 2015-12-10

**Authors:** Lu-Yan Shen, Hui Wang, Bin Dong, Wan-Pu Yan, Yao Lin, Qi Shi, Ke-Neng Chen

**Affiliations:** ^1^ Key Laboratory of Carcinogenesis and Translational Research (Ministry of Education), Department of Thoracic Surgery I, Peking University Cancer Hospital and Institute, Beijing, People's Republic of China; ^2^ Key Laboratory of Carcinogenesis and Translational Research (Ministry of Education), Department of Pathology, Peking University Cancer Hospital and Institute, Beijing, People's Republic of China

**Keywords:** chemotherapy response, ESCC, neoadjuvant chemotherapy, gene expression profiling, MUC

## Abstract

**Background:**

Heterogeneous efficacy of neoadjuvant chemotherapy has led to controversies that have limited its application in clinical practice. Thus, we aimed to identify potential biomarkers predicting esophageal squamous cell carcinoma (ESCC) chemo-responsiveness by gene expression profiling.

**Methods:**

CCK8 assay was used to evaluate the growth inhibitory effect of different concentrations of cisplatin and paclitaxel on the ESCC cell lines EC109, KYSE450, KYSE410, KYSE510, and KYSE150 to differentiate between chemosensitive and chemoresistant cell lines. Gene expression profiling and Real-time PCR were applied to analyze and validate the gene expression differences between chemosensitive and chemoresistant cell lines. IHC was conducted to examine the expression of selected target markers MUC4, MUC13, and MUC20 in 186 ESCC resection samples and the relationships between their expression and tumor regression grade was analyzed. Moreover, RNAi was conducted to instantly block the expression of MUC4, MUC13, and MUC20 to observe their influences on chemo-responsiveness.

**Results:**

EC109 was found to be relatively sensitive to both cisplatin and paclitaxel, while KYSE410 was relatively resistant to cisplatin, KYSE510 was relatively resistant to paclitaxel. Gene expression profiling analysis showed that 2018 genes were differentially expressed in sensitive cell line compared to resistant cell lines. The expression patterns of MUC4, MUC13, MUC20 were validated. Low expression of MUC4 and MUC20 in resection samples was significantly correlated with better TRG. Blockage of MUC20 and MUC13 decreased the drug-resistance capacity and chemosensitivity, respectively.

**Conclusions:**

MUC4 and MUC20 were identified as potential biomarkers for predicting the efficacy of neoadjuvant chemotherapy in ESCC patients.

## INTRODUCTION

Esophageal cancer is a common malignancy for which surgery is currently the major treatment approach. However, for locally advanced stage patients, the 5-yr survival rate remains as poor as 30% even after R0 resection. Hence, it is imperative that an additional treatment be administered to support the major treatment approach, with an aim to improve the survival rate.

Neoadjuvant chemotherapy may be effective in this regard, as the administration of therapeutic agents before surgery might improve the prognosis of these patients. Results of the prominent OEO2 clinical trial conducted by MRC were published in *Lancet* in 2002 [1] and updated in *Journal of Clinical Oncology* in 2009 [2]. These results demonstrated that the R0 resection rate was higher in the group of patients treated with neoadjuvant therapy plus surgery than in the group of patients treated with surgery alone. The median survival time of patients administered neoadjuvant chemotherapy was longer than that of patients treated with surgery alone, with the 2-yr survival rate being 43% and 34%, respectively. Initial results of the RTOG8911 trial (USA inter group 113) conducted by Kelsen et al. were published in the *New England Journal of Medicine* in 1998 [3]; these results demonstrated that the patients analyzed did not benefit from neoadjuvant chemotherapy, but their undated data otherwise showed that prognosis of the patients who responded to chemotherapy was better than that of patients treated with surgery alone, with the median survival time being 3 yr and 1.3 yr, respectively [4]. Hence, patients may not always be sensitive to neoadjuvant chemotherapy; in fact, for those who do not respond to chemotherapy, this toxic treatment is of no benefit and could even be a curse. Thus, predicting the efficacy of neoadjuvant chemotherapy to identify chemosensitive patients is an issue that needs to be addressed urgently.

Since 2000, gene microarray technology has been applied to explore the drug-resistance mechanisms of cancer cells towards cytotoxic drugs. Kudoh et al. [5]. compared differences in gene expression at the transcription level in adriamycin-sensitive and adriamycin-resistant MCF-7 cells and identified potential key genes in cytotoxic drug-resistant cells. Since then, researchers have successively reported the responsiveness of tumor cells to drugs at the transcription level. Previously, researchers have conducted gene expression profiling analysis on pretreatment gastroscopic biopsy specimens of esophageal cancer (most were adenocarcinoma) and attempted to identify genes that could be used to predict sensitivity to neoadjuvant chemoradiotherapy [6–10].

In the current study, we conducted gene expression profiling analysis on 5 esophageal squamous cell carcinoma (ESCC) cell lines that responded differentially to chemotherapeutics to identify neoadjuvant chemo-responsiveness associated genes. Real-time PCR was performed to validate the differentially expressed genes identified in gene expression profiling analysis as targeted markers were selected. IHC was then conducted to assess their expression in resection samples to analyze their relationship with tumor regression grade (TRG), which could reflect chemo-responsiveness, and to evaluate their applicability in predicting neoadjuvant chemosensitivity.

## RESULTS

### Sensitivity of cell lines to Cis-platinum and Paclitaxel

**Figure 1:**
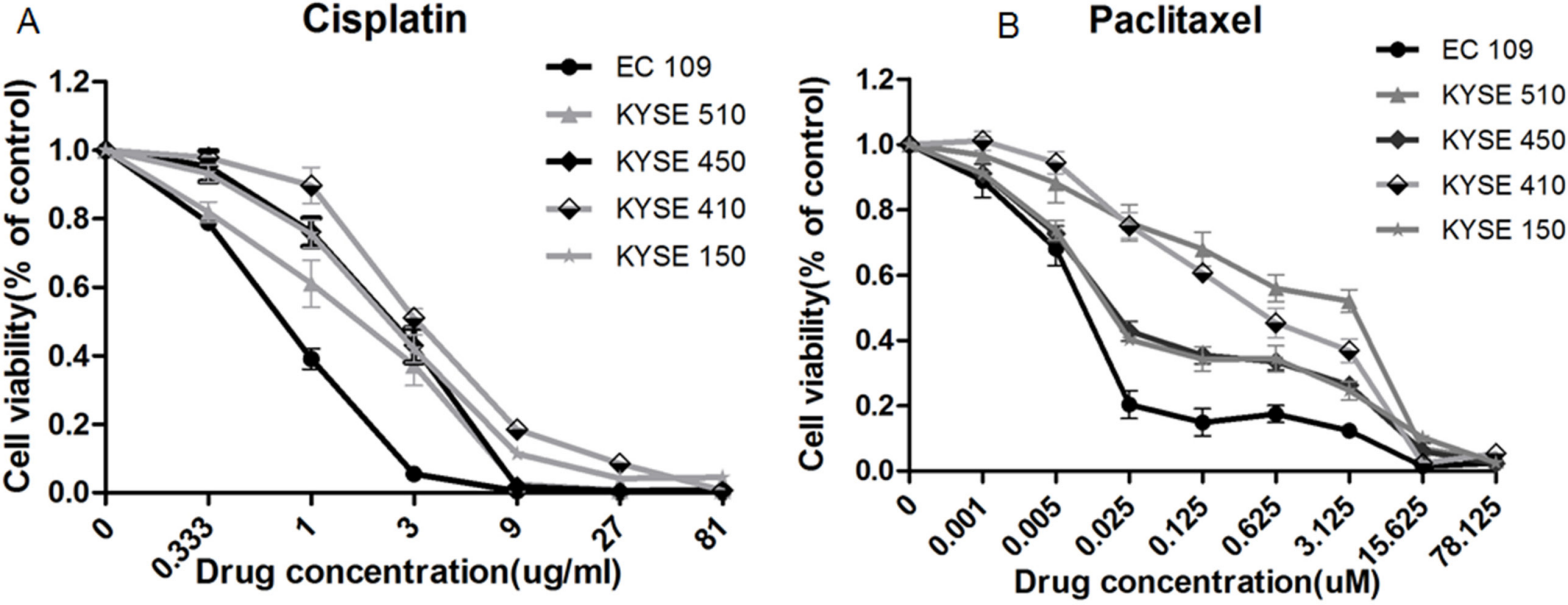
Response of panel of 5 cell lines to cis-platinum and paclitaxel-induced growth inhibition Cells were treated with gradient dilution of drugs for 48 h, the apoptosis was detected using CCK8 assay. (**A**) EC109 cell line was most sensitive to cis-platinum, with IC50 = 0.98 μg/ml, and KYSE 410 was relatively resistant, with IC50 = 3.68 μg/ml; (**B**) EC109 cell line was most sensitive to paclitaxel, with IC50 = 0.0083 uM, and two cell lines of KYSE410 and KYSE510 were relatively resistant, with IC50 = 3.21 uM.

The panel of five cell lines was featured for response to cis-platinum and paclitaxel-induced apoptosis by using CCK8 assay. Cells were treated with gradient concentration of agents and for a period of 48 h, each time in quadruplicate. The result showed that EC109 cell line was relatively sensitive either to cis-platinum or paclitaxel, two of cell lines were relatively resistant (KYSE410 and KYSE510) to paclitaxel and cis-platinum, the remaining three cell lines exhibited intermediate sensitivity to these two agents (Figure 1).

### Microarray analysis and validation

**Figure 2:**
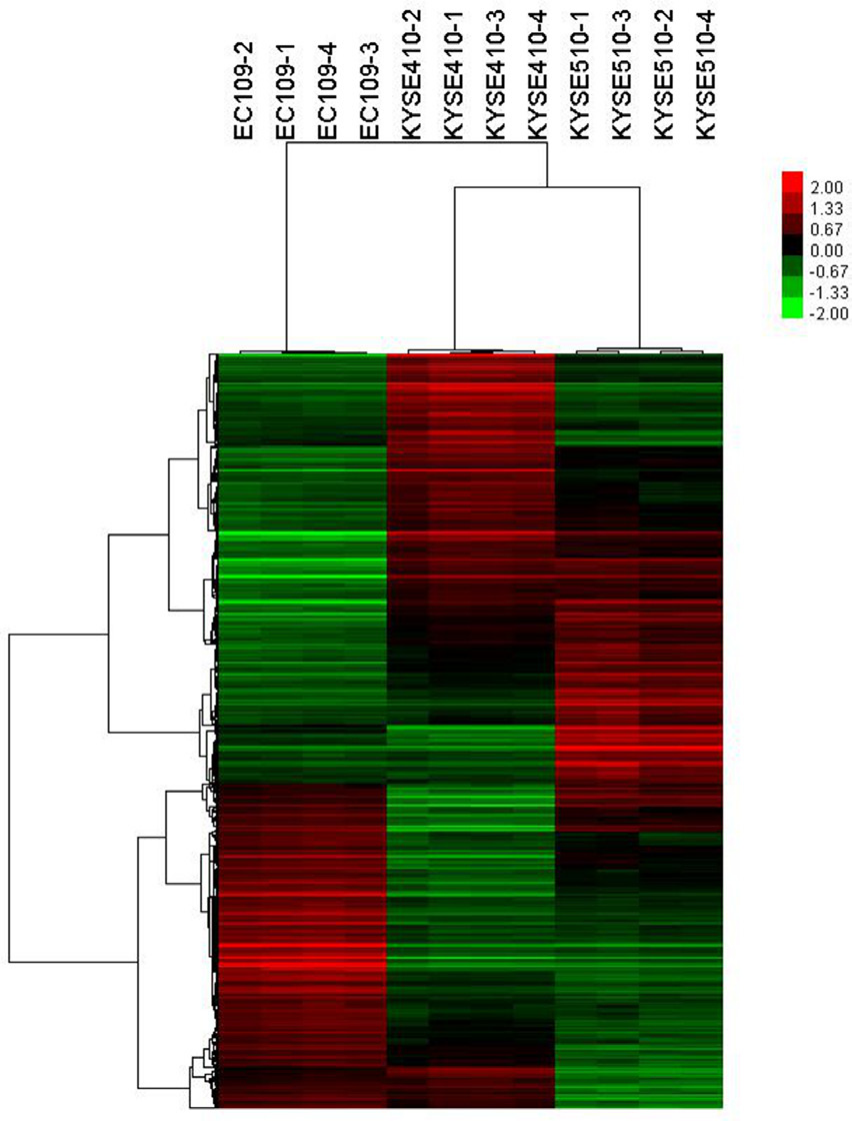
Hierarchical cluster analysis showing differentially expressed genes in cell lines The rows and columns represent genes and cell lines, respectively. The color scale at the top indicates the relative expression levels in terms of standard deviations from the median.

Microarray were used to analyze the gene expression profile in ESCC cell lines. Then, the difference between the relatively sensitive and resistant cell lines on the basal level of expression of each gene was observed. To investigate the genetic variance between cell lines, intensity of each gene in sensitive cell line was divided by that in resistant cell line, and the absolute value of ratio ≥ 1.8 was defined as difference threshold. It was showed that significantly differential expression of 1446 genes was observed in EC109 compared with KYSE410; significantly differential expression of 1386 genes was observed in EC109 compared with KYSE510 (Figure 2). As the combination of multiple agents is becoming increasingly common, to identify gene expression profiles predictive of response to multiple agents could be more useful to make clinical decisions regarding the specific combination of therapies. Therefore, the union of these two sets of differentially expressed genes was used as final results. And a total of 2081 genes were differentially expressed between the relatively sensitive and resistant cell lines.

**Figure 3:**
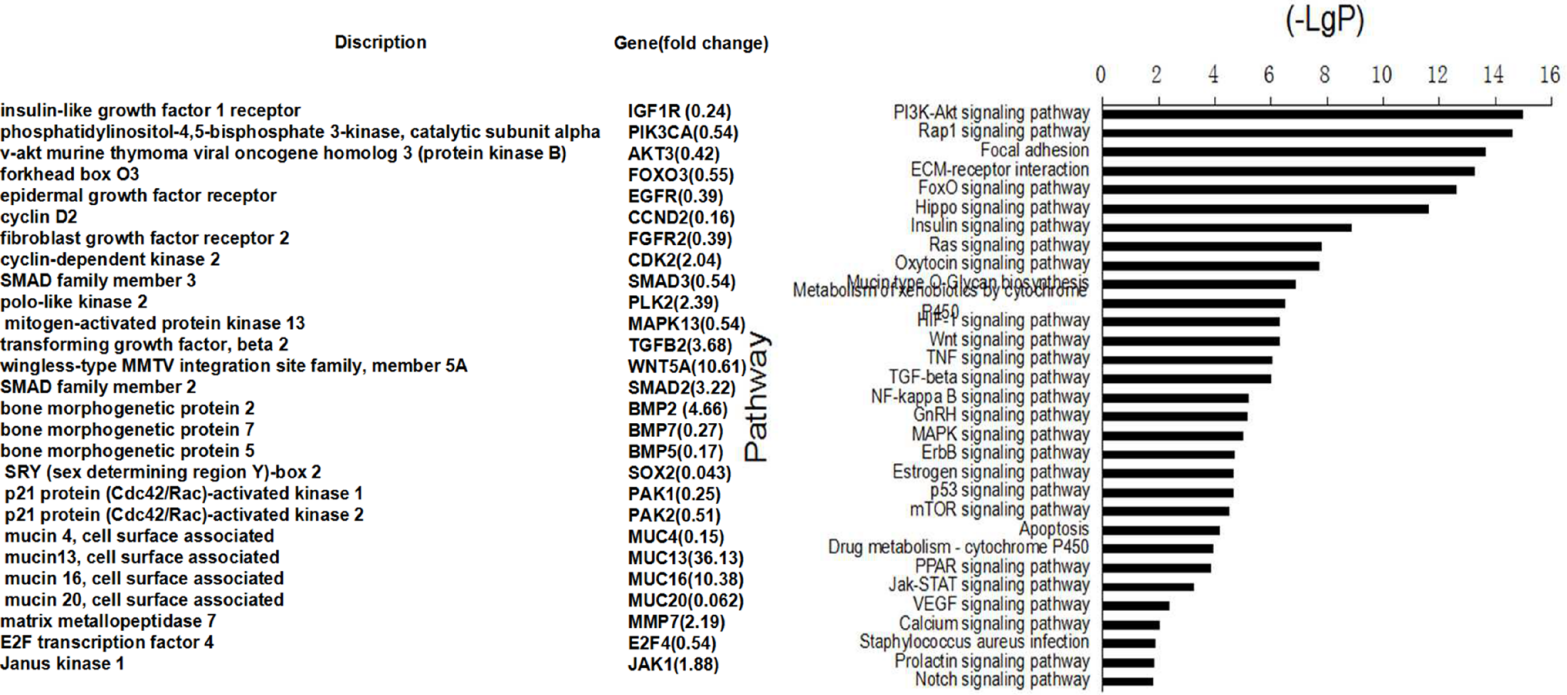
Pathway analysis showed that the differentially expressed genes were involved in the signaling pathways important to cell proliferation, apoptosis, cell adhension and so on (right) The left of figure presented the identified genes and the fold changes in the sensitive cell line vs. resistant cell lines. < 1 indicates downregulation, and > 1 indicates upregulation.

To determine whether these identified genes play a role in specific biological processes, we performed a pathway-net analysis. We found that genes involved in processes, including PI3K-Akt signaling pathway, Mucin type O-glycan biosynthesis, TGF-β signaling pathway, mTOR signaling pathway, were enriched in the sets of genes correlated with chemotherapy agents response (Figure 3).

**Figure 4:**
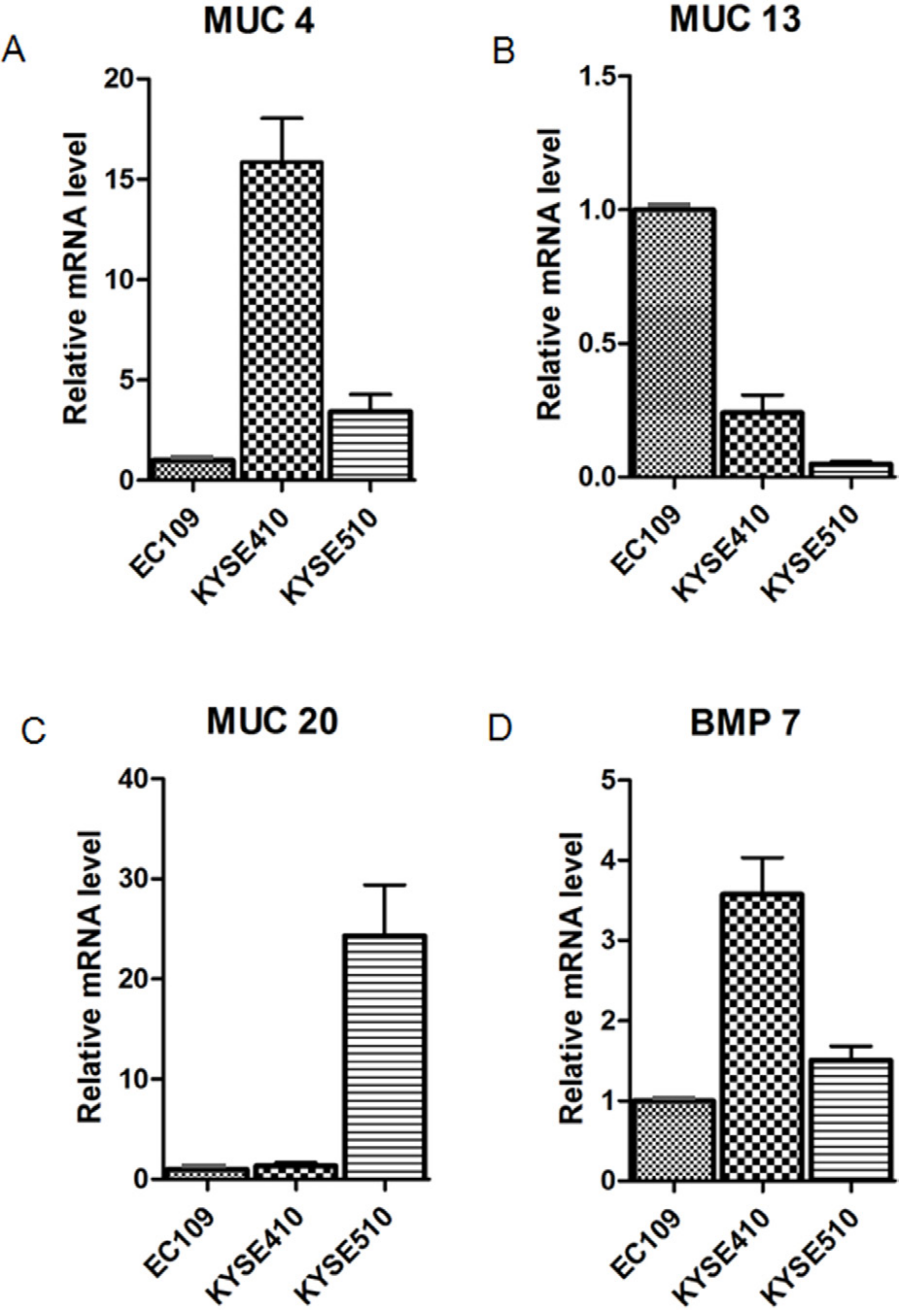
Validation of microarray data analysis The expression of these 6 genes (MUC4, MUC13, MUC20, BMP7, SMAD3, and Akt3) in the panel cell lines (EC109, KYSE410, KYSE510) was measured by qRT-PCR using the 2-ΔΔCt method. The 4 genes shown in the graph were found to be significantly differentiated expressed between sensitive cell line (EC109) and resistant cell lines (KYSE410 and KYSE510) and the results are consistent with the microarray data analysis. Data are expressed as the mean and SD, with relative fold expression on the *y*-axis.

To confirm the microarray data, 6 differentially expressed genes which were involved with the signaling pathways important to carcinogenesis were selected, and their difference in expression across the panel of 3 cell lines (EC109, KYSE410, KYSE510) was confirmed by quantitative real-time PCR. The expression trends 4 of these 6 genes in real-time PCR were consistent with the results from microarray analysis (Figure 4).

### MUC expression was significantly associated with TRG

**Figure 5:**
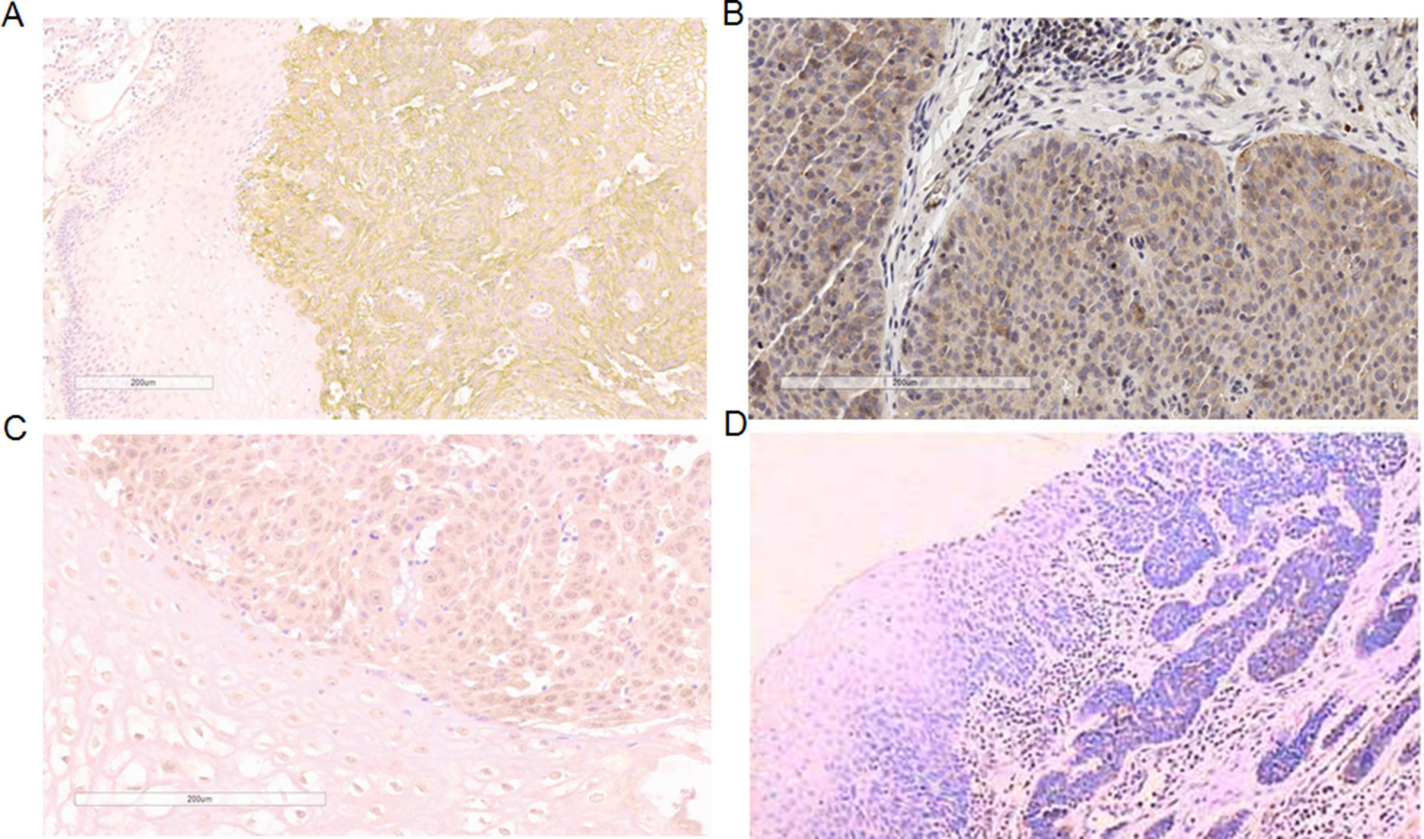
Expression of MUC4/MUC13/MUC20 protein in ESCC (**A**) high expression of MUC4 in ESCC (×200); (**B**) high expression of MUC13 in ESCC (×200); (**C**) high expression of MUC20 in ESCC (×200) (**D**) negative control (×200), with primary antibody replaced by PBS.

To confirm the predictive value of the genes for response to cis-platinum and paclitaxel, we detected their expression on protein level with resected tumor tissue. MUC4 and MUC20 expression were mainly located in cytoplasm (Figure 5), and found in positively 62.4% (116/186) and 38.2% (71/186) of cases, respectively. There was significant correlation between expression of MUC4 and MUC20 with TRG in postoperative specimens. Patients with low expression of MUC4 or MUC20 would have a good tumor regression and fewer residual cancer cells (Tables 1 and 2).

**Table 1:** Association of MUC4 expression in postoperation specimens and tumor regression grade (*n* = 186)

	TRG No. (%)		*P* value
	TRG 1/2/3	TRG4	
Low expression	46 (46.5)	24 (27.6)	
			0.008
High expression	53 (53.5)	63 (72.4)	

**Table 2:** Association of MUC20 expression in postoperation specimens and tumor regression grade (*n* = 186)

	TRG No. (%)		*P* value
	TRG 1/2/3	TRG4	
Low expression	69 (69.7)	46 (52.9)	
			0.018
High expression	30 (30.3)	41 (47.1)	

### MUC expression was associated with chemosensitivity in ESCC

**Figure 6:**
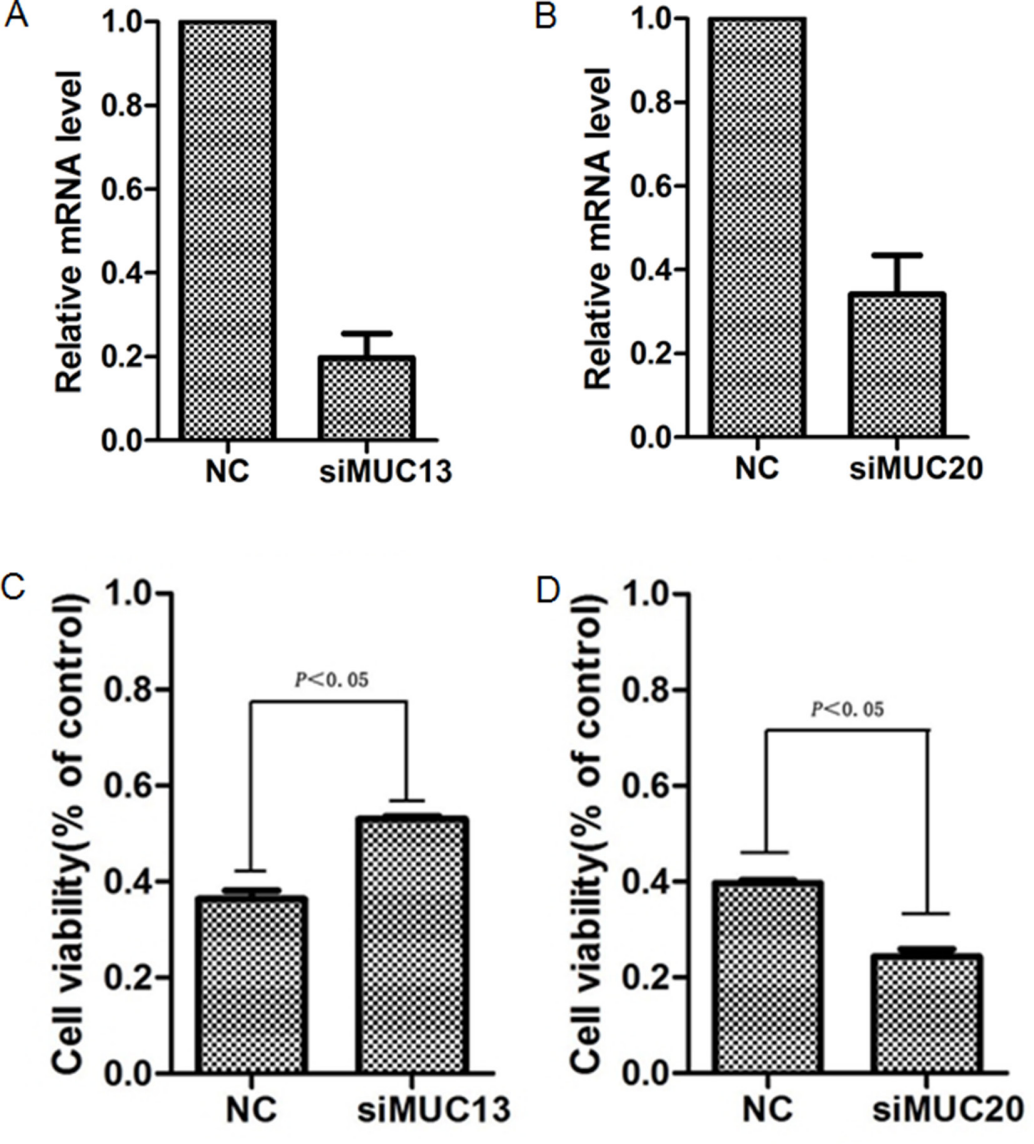
Kncokdown of MUC13 or MUC20 affected the sensitivity of ESCC cells to chemotherapy agent Cells were transfected with either siRNA targeting MUC13, MUC20 gene or scrambled sIRNA for 48 h, then the expression of MUC13 and MUC20 were measured by using Real-time PCR. (**A**) Knockdown of MUC13 in ESCC cell line EC109(sensitive cell line); (**B**) Knockdown of MUC20 in ESCC cell line KYSE510 (resistant cell line). After transient transfection, the cells were treated with IC50 concentration of paclitaxel (0.0083 uM for EC109 and 3.21 uM for KYSE510) in 100 μl medium for 48 h and then the cell viability was measured by using CCK8 assay. (**C**) blockage of MUC13 in EC109 inhibited paclitaxel-induced apoptosis and promoted cell survival. (**D**) blockage of MUC20 in KYSE510 increased paclitaxel-induced apoptosis and the cell viability decreased.

In consideration of the downregulation of MUC4 and MUC20 in relatively sensitive cell line EC109, as well as the upregulation of MUC13 in relatively sensitive cell line EC109, we hypothesized that MUC may be involved in chemotherapy response. To further investigated the effects of MUC on chemotherapy response, we developed a transient cell strain with konckdown of MUC4, MUC13 or MUC20 by transient transfection with siRNA targeted toward MUC4, MUC13 or MUC20, respectively. Real-time PCR showed that MUC4, MUC13 or MUC20 expression were obviously decreased. Then, the cells were treated with the IC50 concentration of cis-platinum or paclitaxel and the cell viability were measured. Knockdown of MUC13 in sensitive cell line EC109 resulted in increased cell survival and inhibited chemosensitivity, whereas, knockdown of MUC20 in resistant cell line KYSE510 promoted cell apoptosis and induced cells more chemosensitive to agent (Figure 6). However, knockdown of MUC4 did not affect cell chemosensitivity.

## DISCUSSION

In this study, we aimed to identify neoadjuvant chemo-responsiveness-associated genes by conducting gene expression profiling analysis on 5 ESCC cell lines. We demonstrated the differences in the sensitivity to both cis-platinum and paclitaxel among the cell lines, and gene expression profiling analysis showed the differences in gene expression patterns of chemosensitive and chemoresistant cell lines. Moreover, the relationship between expression levels and TRG and the effect of blocking gene expression on drug-resistance capacity were elucidated.

Neoadjuvant chemotherapy plus surgery has become the standard treatment approach for locally advanced esophageal cancer. However, the heterogeneous efficacy of neoadjuvant chemotherapy among esophageal cancer patients has impeded its widespread applicability. Moreover, while the long-term survival of chemosensitive patients improves, for chemoresistant patients, neoadjuvant chemotherapy not only fails to improve long-term survival, it could also be a curse for some patients who had a poorer prognosis than patients treated with surgery alone. Thus, predicting the efficacy of neoadjuvant chemotherapy and identifying chemosensitive patients to avoid over-treatment of chemoresistant patients are vital.

In recent years, researchers have tried to identify indicators that could be used to predict the efficacy of neoadjuvant chemotherapy so that these indicators can be considered biomarkers; however, despite these research efforts [12–21], no unanimously recognized molecular biomarkers are currently available for use in clinical practice. Along with the development of high-throughput gene sequencing technology, genetic tests have been widely applied to screen for genes associated with complicated diseases and to predict the efficacy of treatment and estimate the prognosis of the disease.

The current study conducted gene expression profiling analysis in multiple ESCC cell lines possessing different chemo-responsiveness by using cDNA array technology. The obtained results showed that many genes were differentially expressed, among which the expression of more than 200 genes was differentiated, with more than 5-fold difference in expression being observed. Pathway-net was used to analyze the signal pathways in which the differentially expressed genes were involved. The results showed that the number of pathways involving differentially expressed genes was as many as 178, among which 31 signal pathways were associated with the development and progression of cancer (17.4%). This indicates that the genetic information in chemosensitive and chemoresistant cell lines was indeed different.

We selected 6 genes (MUC4, MUC13, MUC20, BMP7, AKT3, and SMAD3) that were closely associated with the development and progression of cancer to validate the conclusions drawn from the gene expression profiling analysis. The results showed that the observed mRNA expression of 4 genes (MUC4, MUC13, MUC20, and BMP7) in chemosensitive and chemoresistant cell lines was in concordance with the results of the gene expression profiling analysis, indicating that the results of the gene expression profiling analysis are reliable. We further validated the expression of MUC4, MUC13, MUC 20, and BMP7 at the mRNA level in the tumor tissues of 2 chemosensitive and 3 chemoresistant ESCC patients before chemotherapy; the results showed that MUC4, MUC20, and BMP7 were expressed at low levels, while MUC13 was over-expressed in the tumor tissues of patients who responded to chemotherapy. This was consistent with the results obtained from the experiments conducted using cell lines. Based on these findings, we considered the MUC family members as potential biomarkers and evaluated their protein expression in 186 paraffin-embedded tumor tissue samples obtained from patients who were treated with neoadjuvant chemotherapy and surgery and analyzed the relationship between their expression levels and TRG. The results demonstrated that MUC4 and MUC20 were expressed at low levels in the tumor tissue samples obtained from chemosensitive patients (TRG1/2/3), whereas the expression of MUC13 did not significantly correlate with TRG. In our previous study, we had analyzed the relation between the expression of MUC memebers and the prognosis of this set of 186 patients and found that the patients with high MUC13/low MUC20 expression had longer median survival time. Since only patients with better chemotherapy response could benefit from neoadjuvant chemotherapy, theoretically, they would have longer survival time. In this study, the patients with better TRG had low MUC20 expression, meanwhile, these patients had favourable prognosis.

Mucins, a group of high-molecular weight transmembrane O-linked glycoproteins secreted by epithelia, are abnormally expressed in many malignancies [22]. Owing to their high molecular weight, the fact that they are heavily glycosylated, and their cell membrane location, mucins could play a role in protecting the cells by acting as a barrier and preventing external substances including anti-tumor drugs from entering the cells. Therefore, mucins have been thought to be potential targets in drug resistance-related research. Bafna et al. [23]. found that the MUC4 gene could inhibit apoptosis and promote cell proliferation by activating the anti-apoptosis pathways, which resulted in the development of gemcitabine resistance in pancreatic cancer patients. The anti-apoptotic effect of MUC4 was demonstrated by the phosphorylation of kinases regulated by her2/extracellular signals and the inactivation of the proapoptotic protein Bad. Hu et al. [24]. found that compared to melanoma cell lines showing a low expression of MUC4, cell lines overexpressing MUC4 were resistant to various chemotherapeutics such as paclitaxel, adriamycin, and vinblastine. The possible underlying mechanism is that the expression of MUC4 could inhibit apoptosis by the activation of caspase-9, which further resulted in the development of drug resistance in tumor cells. To our knowledge, currently, there are no studies on the applicability of MUC family genes to the prediction of neoadjuvant chemosensitivity in ESCC patients. We found that MUC4 and MUC20 were expressed at low levels, and MUC13 was over-expressed in chemosensitive ESCC cell lines. By the *in vitro* experiment, we found that blocking of MUC4 and MUC20 could contribute to the increase in the sensitivity of ESCC cell lines to paclitaxel, whereas blocking of MUC13 resulted in a decrease in the sensitivity of ESCC cell lines to paclitaxel. These results further indicate that the MUC family genes are potential biomarkers in predicting the efficacy of chemotherapy for ESCC patients.

Although some studies have already reported the applications of gene expression profiling analysis in identifying neoadjuvant chemosensitivity-associated biomarkers of ESCC, the current study puts forth some novel findings: 1. Most of the studies enrolled patients treated with neoadjuvant chemoradiation and surgery instead of patients treated with neoadjuvant chemotherapy and surgery. 2. The pathological type of the cancer in the patients was adenocarcinoma; therefore, information with respect to esophageal squamous cell carcinoma is lacking. To our knowledge, only one study by Motoori [25] evaluated neoadjuvant chemotherapy in ESCC, in which gene expression profiling analysis was performed using pretreatment fresh biopsy specimens obtained from 25 ESCC patients who received neoadjuvant therapy and the neoadjuvant chemotherapy responsiveness-predicting model, which involved 199 differentially expressed genes, was constructed. It is noteworthy that some differentially expressed genes such as TCEA3, IFI6, and PEP overlapped between the two studies; this aspect needs to be further validated in the future.

The current study also has some limitations. Owing to the difficulties in procuring large biopsy specimens before surgery, we were left with no choice but to use postoperative paraffin-embedded samples for our clinical validation. Therefore, it is uncertain whether chemotherapy could change the expression of these genes. Despite the shortcomings of the study, the results obtained still amount to powerful evidence regarding molecular biomarkers that could be used to predict the efficacy of neoadjuvant chemotherapy in ESCC patients. In the future, further prospective studies are needed to confirm these findings.

## MATERIALS AND METHODS

### Cell lines and cell culture

Esophageal squamous cell lines KYSE410, KYSE510, KYSE450, KYSE150, and EC109 was cultured in RPMI1640 (Hyclone) medium with 10% heat-inactivated fetal bovine serum in a humidified atmosphere with 5% CO_2_, at 37°C.

### CCK8 assay

Cells were plated in 96-well plates at a density of 10,000 cells per well in 100 μl of complete medium and grown overnight. Then the cells were treated with gradient dilution of cis-platinum (0.333, 1, 3, 9, 27, 81 μg/ml) and paclitaxel (0.001, 0.005, 0.025, 0.125, 0.625, 3.125, 15.625, 78.125 μM) in 100 μl medium. After 48 h of incubation, a total of 100 μl of Cell Counting Kit-8(CCK8) reagent (Dojindo) was added to each well. After 4 h of incubation at 37°C, the absorbance of per well was measured at 450 nm using a VERS Amax Microplate Reader (Molecular Devices Corp, Sunnyvale, CA).

### Microarray analysis

For Affymetrix microarray profiling, total RNA was isolated with Trizol reagent (Invitrogen, Canada) and purified using RNeasy Mini Kit (Qiagen, German), including a DNase digestion treatment. RNA concentrations were determined by the absorbance at 260 nm and quality control standards were A260/A280 = 1.8–2.1, using NanoDrop 2000 (Thermo, America). cDNA of cell lines KYSE510, KYSE410, and EC109 was hybridized to GeneChip Human Transcriptome Array 2.0 (Affymetrix, America) according to the User Manuals.Affymetrix^®^ Expression Console Software (version 1.2.1) was used for microarray analysis. Raw data (CEL files) were normalized at transcript level using robust multiaverage method (RMA workflow). Median summarization of transcript expressions was calculated. Gene-level data was then filtered to include only those probe sets that are in the ‘core’ metaprobe list, which represent RefSeq genes.

### Quantitative real-time PCR

Total RNA was was reverse-transcribed using the Reverse Transcription System (Thermo scientific com). The reverse transcription reaction was performed sequentially for 60 min at 42°C, and for 5 min at 70°C. Real-time PCR was performed using SYBR Green. PCR runs and fluorescence detection were performed in a Rotor-Gene 6000 Real-Time PCR system (Applied Biosystems). The sequences of the real-time PCR primers were as follows: MUC4: forward 5′-AACGCAAGCATCGGACT TCACAC-3′ and reverse 5′-TAGGCTTCAATCACACGACCACCA-3′, MUC13: forward 5′-ACCAAGATCTGAAATGCGTGCT-3′ and reverse 5′-CTAAACCAT TGAGGCAGTCATCCG-3′, MUC20: forward 5′-CAAGATCACAACCTCAGC GA-3′ and reverse 5′-ACCTCCATTTTCACCTGCAC-3′, BMP7: forward 5′-CCA TTTCCTCACCGACGCCGACA-3′ and reverse 5′-ATCCGATTCCCTGCCCAAG TGCTC-3′, Akt3: forward 5′-TGCCTTGGACTATCTACATTCCG-3′ and reverse 5′-GGCCATAGTCATTATCTTCTAACACC-3′, smad 3: forward 5′-TGCACC ATCCGCATGAGCTTCGT-3′ and reverse 5′-TGCACCATCCGCATGAGCTTCG T-3′. The cycling conditions were as follow: 95°C for 10 min, followed by 40 cycles of 95°C for 20 s, 58°C for 20 s, and 70°C for 20 s. GAPDH was used as an internal control.

### RNA inference and cell transfection

For knock-down of MUC4 MUC13 or MUC20 expression in KYSE510, siRNA was used. The oligonucleotides containing MUC siRNA sequences were synthesized (GenePharma Com). MUC13 siRNA sequences were 5′-GCGUGCUGAUGACAC GUUUTT- 3′ and 5′-AAACUUGUCAUCAGCACGCTT-3′, MUC4 siRNA sequences were 5′-GCUGGAAUGACAAGCCCUATT-3′ and 5′-UAGGGCUUGU CAUUCCAGCTT-3′, MUC20 siRNA sequence were 5′-GCGCCUCACUUCCAG GUCUTT-3′, and 5′-AGACCUGGAAGUGAGGCGCTT-3′. Cells were plated (3 × 105 cells) in a 6-well plate and grown overnight to 50% confluence, and then transfected with 100 μl medium containing siRNA targeting MUC4, MUC20 gene or scrambled siRNA. After 48 h, the total RNA was isolated for real-time PCR.

### Immunohistochemistry

After routine deparaffinization and hydration, tissue sections were treated with 3% hydrogen peroxide and then heated in citrate buffer (pH 6.0) for antigen retrieval. The MUC4 or MUC20 antigen-antibody reaction took place overnight at 4°C, following goat serum blocking. The streptavidin/Peroxidase amplification kit (Zymed) was applied to detect the signal of the MUC4 or MUC20 antigen-antibody reaction. Peroxidase activity was developed with diaminobenzidine. All sections were counterstained with hematoxylin. The purified rabbit polyclonal antibody against human MUC4 or MUC20 (Abcam) was used at 1:200 and 1:100 respectively, and goat anti-rabbit biotin-conjugated IgG was used as secondary antibody. Immunohistochemical signals were scored by two independent observers. The scores were calculated multiplying the staining intensity and extent. The staining intensity was categorized by relative intensity as follows: 0, negative; 1, weak; 2, moderate; and 3, strong. The proportion of cells proteins expression was categorized as follows: 0, < 10% immunopositive cells; 1, 10%–30% positive cells; 2, 30%–60% positive cells; 3, > 60% positive cells. Scores < 6 was considered as low-level expression, whereas scores of 6 or 9 were considered as high-level expression.

### Patients and specimens

Formalin-fixed paraffin-embedded (FFPE) blocks of postoperative specimens of 186 successive ESCC patients who underwent neoadjuvant chemotherapy followed by surgery in a single-surgeon team between January 2000 and December 2012 at the Department of Thoracic Surgery I, Peking University Cancer Hospital were obtained. All of the patients presented ESCCs at stage T2–3N1M0. This study was approved by both the Ethics and the Academic committees of Peking University School of Oncology, and informed consent was obtained from all patients.

### Neoadjuvant chemotherapy

Platinum-based 2-drug combination, mainly the paclitaxel and cis-platinum with the proportion of 95%, was used in neoadjuvant chemotherapy. On day 1, paclitaxel at a dose of 175 mg/m^2^ of body surface area was administered intravenously. On days 1–3, cis-platinum at a dose of 25 mg/m^2^ of body surface area was administered intravenously, a single course of treatment lasted 21 d. Enhanced chest computed tomography (CT) and esophagography were used to evaluate the curative effects of the treatment. Approximately 1–4 cycles of neoadjuvant chemotherapy were administered before surgery. Among them, 64.6% received 2 cycles (120/186).

### Tumor regression grade assessment

All enrolled subjects were reviewed again by two experienced pathologists who were blinded to the clinical information and gene expression. Tumor regression grade (TRG) was graded on 4-point scale based on the histologic tumor response assessment described by Mandard [11]. This assessment was defined as: grade I, no residual tumor cells; grade II, nearly complete response with < 10% vital residual tumor cells (VRTC); grade III, 10%–50% VRTC; and grade IV, > 50% VRTC.

### Statistical analysis

SPSS 19.0 software was used to perform the statistical analyses. All *in vitro* experiments were performed at least thrice in triplicates. When the data from different groups were compared, normal analysis and homogeneity of variance were checked first, and then an unpaired two-tailed *t* test analysis was used. Bars and error bars on the graphs as well as data in the text represent the mean ± SD. The correlation between gene expression and tumor regression grade (TRG), as well as between gene expression and clinicopathological downstage was evaluated by chi-squared test.

## SUPPLEMENTARY MATERIALS FIGURE, TABLE


