# Supplementary material for: Possible prediction of the response of esophageal squamous cell carcinoma to neoadjuvant chemotherapy based on gene expression profiling
Source: Oncotarget. 2015 Dec 10;7(4):4531–41. doi: 10.18632/oncotarget.6554 (PMC4826224; doi:10.18632/oncotarget.6554)
Supplement: Supplementary file 1 [file oncotarget-07-4531-s001.pdf]

Possible prediction of the response of esophageal squamous cell carcinoma to neoadjuvant chemotherapy based on gene expression profiling

Supplementary Materials

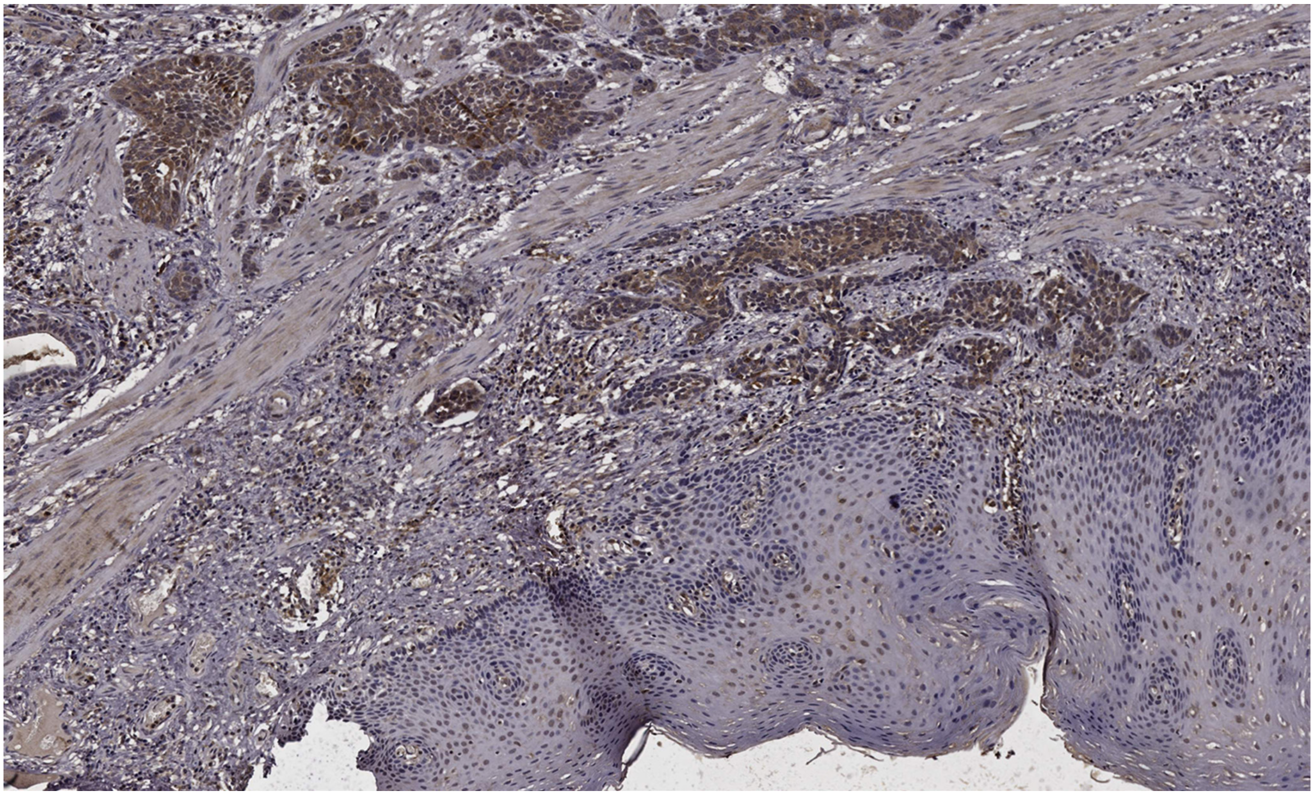

Supplementary Figure S1: High expression of BMP7 protein in ESCC cells but low expression in noncancer epthelium cells (x100).

Supplementary Table S1: Association of BMP7 expression in postoperation specimens and tumor regression grade (*n* = 186)

Item	TRG		*P* value
	TRG 1/2/3	TRG4	
BMP7 expression			
Low	35 (35.4)	21 (24.1)	0.096
High	64 (64.6)	66 (69.9)	
